# Editorial: Community series in frozen elephant trunk surgery in aortic dissection: volume II

**DOI:** 10.3389/fcvm.2024.1504544

**Published:** 2024-10-22

**Authors:** Bleri Celmeta, Amer Harky, Antonio Miceli

**Affiliations:** ^1^ Minimally Invasive Cardiac Surgery Unit, IRCCS Ospedale Galeazzi - Sant'Ambrogio, Milan, Italy; ^2^ Department of Cardio-Thoracic Surgery, Liverpool Heart and Chest Hospital, Liverpool, United Kingdom

**Keywords:** aortic dissection, frozen elephant trunk (FET), intraluminal stent thrombosis, conventional technique, branch priority

**Editorial on the Research Topic**
Community series in frozen elephant trunk surgery in aortic dissection: volume II

## Introduction

Acute aortic dissection (AD) remains a serious condition requiring emergent medical attention. Mortality remains very high, approaching 50% in the first 48 h without surgical repair (1), or 1%–2% per hour until surgery is performed (2). In contrast, in-hospital mortality has gradually fallen from 31% to 22%, thanks to improved surgical outcomes (3, 4). A previous Research Topic (5) reviewed the evolution of the frozen elephant trunk (FET) approach to the treatment of AD and discussed some of the key technical and technological advances. The current collection now explores additional aspects of FET in AD. According to recent ESC guidelines for the management of aortic disease, FET may be considered in patients with acute AD and a secondary intimal tear in the arch or proximal descending thoracic aorta, but there is as yet no evidence to strongly recommend this strategy (1). In addition, our growing experience of short- and long-term follow-up of patients who have undergone FET enables us to better explore its benefits and its most frequent complications. Such is the case of intraluminal stent thrombosis (ILT), which was initially under-diagnosed.

The articles of this collection provide a valuable contribution to the search for the most appropriate surgical strategy when faced with emergent acute AD in the operative theatre (Table 1).

**Table 1:** Summary of the collection of the articles.

Authors	Title	Keywords	Summary
Göbel et al.	Frozen elephant trunk versus conventional proximal repair of acute aortic dissection type I	• Aortic dissection • Frozen Elephant Trunk • Conventional repair	The authors found no difference in the mortality between FET and conventional repair after acute AD. However, FET patients experienced fewer strokes (2.8% vs. 11%, *p* = 0.03) and similar rates of spinal cord injury.
Wisniewski et al.	The frozen elephant trunk in acute aortic dissection: an ultimate solution? Institutional experience	• Aortic dissection • Frozen Elephant Trunk • Monocentric	The outcomes after three different surgical techniques for acute AD (FET, total arch replacement and hemiarch replacement) were explored in this monocentric study. The FET group showed a lower early mortality rate and higher mid-term survival rates. However, the use of different surgical approaches was not found to be a predictor of mortality.
Gao et al.	Aortic arch branch-prioritized reconstruction for Type A aortic dissection surgery	• Aortic dissection • Branch-prioritized repair • Conventional repair	When compared with the classical surgical technique, the branch-priority technique was associated with significantly reduced cardiopulmonary bypass time, shorter ICU stay due to shorter ventilation time and earlier wake-up time and lower incidence of neurological complications, promoting faster postoperative recovery.
Wang et al.	FL% is associated with the severity of acute DeBakey type I aortic dissection in patients undergoing frozen elephant trunk and total arch replacement	• Aortic dissection • Ascending aorta FL% • Severity index	The ascending aortic FL%, the location of the initial tear (more severe if initial tear in ascending aorta), and the involvement of the left iliac and right coronary arteries were found to be significant predictors of acute aortic dissection severity.
Helms et al.	Risk factors, prevention, and therapy of intraluminal stent thrombosis in frozen elephant trunk prostheses -what we know so far	• Frozen elephant trunk • Intraluminal stent thrombosis • Stent thrombosis management	Intraluminal stent thrombosis following FET, occurring in 6%–17% of patients, is associated with increased morbidity and mortality. Early diagnosis is essential in order to address properly this highly relevant complication and to avoid postoperative embolic events.

FET, frozen elephant trunk; AD, aortic dissection.

## Papers presentation

The best surgical treatment for acute type A aortic dissection is still debated. This is why in their article, Göbel et al. compared the outcomes of the frozen elephant trunk (FET) procedure to conventional proximal repair for treating type I acute aortic dissection. They examined 172 patients who underwent emergent surgery between 2009 and 2016, with 72 receiving FET and 100 undergoing conventional repair. The study focused on 30-day mortality, neurologic deficits, and long-term follow-up data. Key findings showed no significant difference in early or late mortality between the two groups. However, FET patients experienced fewer strokes (2.8% vs. 11%, *p* = 0.03) and similar rates of spinal cord injury. FET surgery is generally associated with distal aortic remodeling and false lumen thrombosis. But surprisingly, in this study, aortic-related reintervention rates were comparable. The explanation might be found in the reduced number of open distal descending aorta surgery on the conventional group due to the higher surgical risk, and the higher number of distal endovascular extension after FET whenever indicated, being this approach safer than the open strategy.

In their study, Wisniewski et al. described their Institutional experience of surgical treatment of acute aortic dissection, comparing the early and mid-term results after three different surgical techniques: FET, total arch replacement (TAR) and hemiarch replacement (HR). After including 213 patients treated between April 2015 and May 2023, the FET group showed a lower early mortality rate (8.9%) compared to the TAR (33%) and HR (17%) groups, despite similar preoperative characteristics and higher GERAADA score in FET group (6). However, the use of different surgical approaches was not found to be a predictor of mortality. Patients treated with FET also had better mid-term survival rates, with an overall mortality of 24.4%, compared to 45.4% for TAR and 34.1% for HR. The authors concluded that although more complex and technically demanding, FET is a viable and effective treatment in AD as it may lead to improved early and mid-term survival rates. Further research is needed to refine techniques and address limitations.

The study by Gao et al. investigated the efficacy of an aortic arch branch-prioritized reconstruction technique for Stanford type A aortic dissection (STAAD) surgery. Briefly, the supra-aortic vessels were reconstructed before the aortic replacement, by establishing a right femoro-subclavian arterial autologous bypass. After the occlusion of the innominate artery, the cerebral perfusion was guaranteed by the femoral artery, allowing the reconstruction of the supra-aortic vessels by mean of a Y-shaped graft, which was subsequently anastomosed termino-laterally to the ascending aorta Dacron prosthesis. This technique allowed to reduce CPB times and to perform complete bilateral cerebral perfusion during cardiac arrest with higher body temperatures. The study included 97 patients undergoing total arch replacement and frozen elephant trunk procedures between 2018 and 2021. Patients were divided into two groups: 35 underwent the branch-priority technique, while 62 had the classic method. The branch-priority group showed significantly reduced cardiopulmonary bypass time, shorter ICU stay due to shorter ventilation time and earlier wake-up time and lower incidence of neurological complications, promoting faster postoperative recovery.

In the study from Wang et al. a new parameter was introduced by the authors. FL% was defined as the ratio of the false lumen perimeter to the total aortic (true lumen + false lumen) perimeter at different levels of the aorta. This parameter can be easily measured in the preoperative CT scan. After analyzing 344 patients treated from 2015 to 2019, dividing them into high-risk and ultra-high-risk groups based on perioperative severity, the results showed that ascending aortic FL%, location of the initial tear (more severe if initial tear in ascending aorta), and involvement of the left iliac and right coronary arteries were significant predictors of disease severity. Thus, the study concluded that ascending aorta FL% may be a reliable radiological indicator for assessing the extent of aortic dissection and could guide treatment strategies. However, larger multicenter studies are recommended to confirm these findings and further evaluate the use of FL% in clinical practice.

In their paper, Helms et al. conducted a thorough review of the literature describing various aspects of intraluminal stent thrombosis (ILT) following FET, a highly relevant complication which is receiving the deserved attention only recently. ILT is defined as an endograft thrombus with minimum size of 2 × 4 mm. With an incidence of 6%–17% of patients receiving FET, it is associated with increased morbidity and mortality due to embolic events. The review discussed the risk factors associated with ILT, including thoracic aneurysm and its diameters, the stent-to-aneurysm diameter ratio, the perioperative coagulation management and hyper-coagulable state. Preventive strategies focus on aggressive anticoagulation management, including the importance of meticulous surgical hemostasis to avoid intraoperative usage of pro-coagulation products, and optimizing stent graft designs to reduce low-flow zones. For existing ILT cases, prolonged anticoagulation or endovascular interventions like stent extensions were recommended by the authors. However, the long-term effectiveness of these measures remains to be proven. The article emphasized the importance of early diagnosis and treatment to improve patient outcomes and called for further studies to establish optimal prevention and treatment protocols.

## Conclusion

In conclusion, the articles in this new Research Topic summarize the efforts of the medical and surgical community to improve the technical aspects of FET for the treatment of AD, to define the surgical strategy best suited to the characteristics of the patient and the AD itself, and to explore unknown and potentially fatal postoperative conditions, all with the ultimate objective of improving surgical outcome and postoperative evolution.
